# New methods for computational decomposition of whole-mount *in situ* images enable effective curation of a large, highly redundant collection of *Xenopus* images

**DOI:** 10.1371/journal.pcbi.1006077

**Published:** 2018-08-29

**Authors:** Ilya Patrushev, Christina James-Zorn, Aldo Ciau-Uitz, Roger Patient, Michael J. Gilchrist

**Affiliations:** 1 The Francis Crick Institute, London, United Kingdom; 2 Cincinnati Children’s Hospital, Division of Developmental Biology, Cincinnati, Ohio; 3 MRC Molecular Haematology Unit, The Weatherall Institute of Molecular Medicine, University of Oxford, John Radcliffe Hospital, Headington, Oxford; Max Planck Institute of Molecular Cell Biology and Genetics, GERMANY

## Abstract

The precise anatomical location of gene expression is an essential component of the study of gene function. For most model organisms this task is usually undertaken via visual inspection of gene expression images by interested researchers. Computational analysis of gene expression has been developed in several model organisms, notably in *Drosophila* which exhibits a uniform shape and outline in the early stages of development. Here we address the challenge of computational analysis of gene expression in *Xenopus*, where the range of developmental stages of interest encompasses a wide range of embryo size and shape. Embryos may have different orientation across images, and, in addition, embryos have a pigmented epidermis that can mask or confuse underlying gene expression. Here we report the development of a set of computational tools capable of processing large image sets with variable characteristics. These tools efficiently separate the *Xenopus* embryo from the background, separately identify both histochemically stained and naturally pigmented regions within the embryo, and can sort images from the same gene and developmental stage according to similarity of gene expression patterns without information about relative orientation. We tested these methods on a large, but highly redundant, collection of 33,289 *in situ* hybridization images, allowing us to select representative images of expression patterns at different embryo orientations. This has allowed us to put a much smaller subset of these images into the public domain in an effective manner. The ‘isimage’ module and the scripts developed are implemented in Python and freely available on https://pypi.python.org/pypi/isimage/.

## Author summary

An important component of research into the function of genes in the developing organism is an understanding of both when and where the gene is expressed. Well established molecular techniques can be used to colour the embryo in regions where the gene of interest appears, and researchers will photograph such treated embryos at different stages of development to build up the story of the gene’s use. Small numbers of these *expression pattern* images may easily be examined by eye, but getting usable information from large collections of such images would take an enormous investment in time by trained scientists. Computational analysis is much to be preferred, but the task is complex and difficult to generalise. The frog *Xenopus* is an important model for studying vertebrate development, but up till now has had no purely computational methods available for analysing gene expression. Here we present a suite of computational tools based on a range of mathematical methods, capable of recognising the outline of the embryo against a variety of backgrounds, and within the embryo separately recognising areas of both gene expression and natural pigmentation. These tools work over a wide range of embryo shapes and imaging conditions, and, in our opinion, represent a major step towards full automation of anatomical gene expression annotation in vertebrate embryology.

## Introduction

A significant challenge for current bioinformatics is the computational analysis of large data sets. Recent developments in sequencing technologies have allowed, for example, the investigation of the time course of gene expression in early development of *Xenopus tropicalis* at high time resolution [1,2]. For a robust understanding of gene expression, the precise anatomical or cellular location of expression is as important as the timing of expression, yet this presents significant challenges for computational analysis. The most advanced work has been done in *Drosophila*, with the analysis of the time evolution of the spatial pattern of gene expression revealing genes with co-localised expression[3,4,5,6].

The spatial distribution of RNA within an embryo or tissue is typically obtained by *in situ* hybridisation (WISH) of a probe sequence to the endogenous RNA under study or by protein immunofluoresence, followed by photographic imaging of the required stages and views or sections. Preparation of reagents and optimisation of conditions for a specific protein/gene target may take time, but once done it is straightforward to generate images covering (for example) many different developmental stages.

For studies on the localisation of small numbers of genes, analysis by inspection of the resultant images is likely to be feasible and may provide sufficient descriptive data to answer the biological question at hand. In larger scale screens the number of generated images can grow rapidly to tens of thousands [3] or more [7], and at this level will either require computational analysis or significant commitment by members of the respective model organism community to manually annotate the images; for example with zebrafish [8], *Drosophila* [6] or *Xenopus* [9,10]. However, although manual annotation is generally of high quality, it is slow and the required effort is not easily replicated.

Computational analysis is clearly preferred for large numbers of images, although this is not a straightforward task, and may require significant investment of time and expertise to develop a suitable system. The goals of computational analysis are easily stated: to recognize the relevant physical anatomy of the organism in the image, locate the regions which show gene expression, and either label these regions with suitable anatomical terms or transfer them to a model coordinate system within which the expression patterns may be analysed and/or compared. These goals are usually achieved by two distinct processes described as *segmentation* (recognising compartmentalisation in the image) and *registration* (fitting the embryo shape in the image to a model), as well as recognising which parts of the segmented image correspond to gene expression.

Image analysis in *Xenopus* has several specific challenges: the embryos are not normally fully transparent; embryos may display distinct pigmented regions; in embryos that are cleared to make them transparent the outline of the embryo may merge into the image background; and experimental data frequently cover a wide range of development stages and concomitant variety of embryo shapes and sizes. In addition, the earlier development stages are quasi-spherical, and, unlike fly embryos, may present some difficulty in determining the axial orientation within the image. To date there are no published methods for computational image analysis developed for *Xenopus*.

Here we report a first suite of tools developed for computational analysis of *Xenopus in situ* images. These tools are capable of cleanly separating the embryo from the image background over a wide range of developmental stages without requiring the background to be either uniform or any specific colour; *in situ* hybridisation stain and natural pigment are detected independently and can be marked up accordingly; and analysed images at the early quasi-spherical stages can be compared with each other to identify groups of images photographed at the same axial orientation. Application of this solution of the segmentation problem and partial solution of the registration problem has enabled us to analyse a large and highly redundant image collection, selecting a usefully condensed and representative set for public dissemination. Although it remains to provide the ability to register the images in a model coordinate system, we have laid some useful ground work for future progress. The reduced image set may also now be considered for manual image registration, expression pattern extraction and annotation in existing *Xenopus* community tools such as Xenbase (http://www.xenbase.org, RRID:SCR_003280) [11,12] and XenMARK (https://genomics.crick.ac.uk/apps/XenMARK, RRID:SCR_014924) [9].

Two of us (MG and IP) were motivated to undertake this research by the desire to complement our high resolution time series data in *Xenopus tropicalis* [1,2] with expression localisation data mined from public image collections, and to promote and enable further work on computational image analysis within the community. Earlier work [9] had suggested a way forward through crowd sourcing of manual annotation, but the generation, and donation to the community, by others of us (AC-U and RP) of a large collection of 33,289 informative *in situ* images, suggested that we consider computational approaches. This large set of images contained multiple images at given developmental stages for each gene, and we reasoned that a systematic reduction of this (around 10-fold) technical redundancy would yield a more useful and tractable set of images for use by other researchers via submission to Xenbase, the *Xenopus* the model organism database. Computational tools devised to achieve this would necessarily form a sound basis for further progress in image analysis in *Xenopus*. We do not at this stage provide a solution to the problem of registering embryo outlines with a model representation.

## Results

In this section we present the functional outline and application of each part of our method. More details of the basis of each algorithm are provided in the Methods section below.

In summary, we developed two primary algorithms: (i) embryo-masking through image segmentation to separate the part of the image containing the embryo from the image background without prior constraints on the background colour or texture; and (ii) colour-separation within the embryo outline, to identify the approximate hues of *in situ* stain, and pigmented and un-pigmented embryo in each image, and mark up the image accordingly. In addition, we developed algorithms to automatically classify large sets of images by background characteristics, and to perform image-clustering on spherical stage images under transformations of scale, rotation, and shear and hence identify groups of images for the same gene and stage but photographed at different orientations. This last was a key tool in applying our methods to the large, redundant image collection described above, and may also provide a way forward to a solution of the general registration problem for *Xenopus* embryos.

To develop the methods we have drawn on two sources of *Xenopus* in situ images: firstly, locally hosted images from the XenMARK project[9] covering a wide range of imaging conditions, and second, a collection of 33,289 images provided by two of us (AC-U and RP) which is described in more detail below. Randomly selected images from both these collections were used for validation.

A brief overview of the primary algorithms is given here, with more technical detail presented in Methods §2 and §3.

### Segmentation for embryo masking

This algorithm locates the outline of the embryo within the image. We made two assumptions (i) that the distribution of colour and texture within the embryo is distinct from the distribution of colour and texture in the background, and (ii) that at least part of the embryo is more or less centrally located within the image. These are generally reasonable for the great majority of images we have seen during the development of this work. As a useful side-effect we can also detect images where we believe the embryo touches or is intersected by the edges of the image frame.

**Fig 1:**
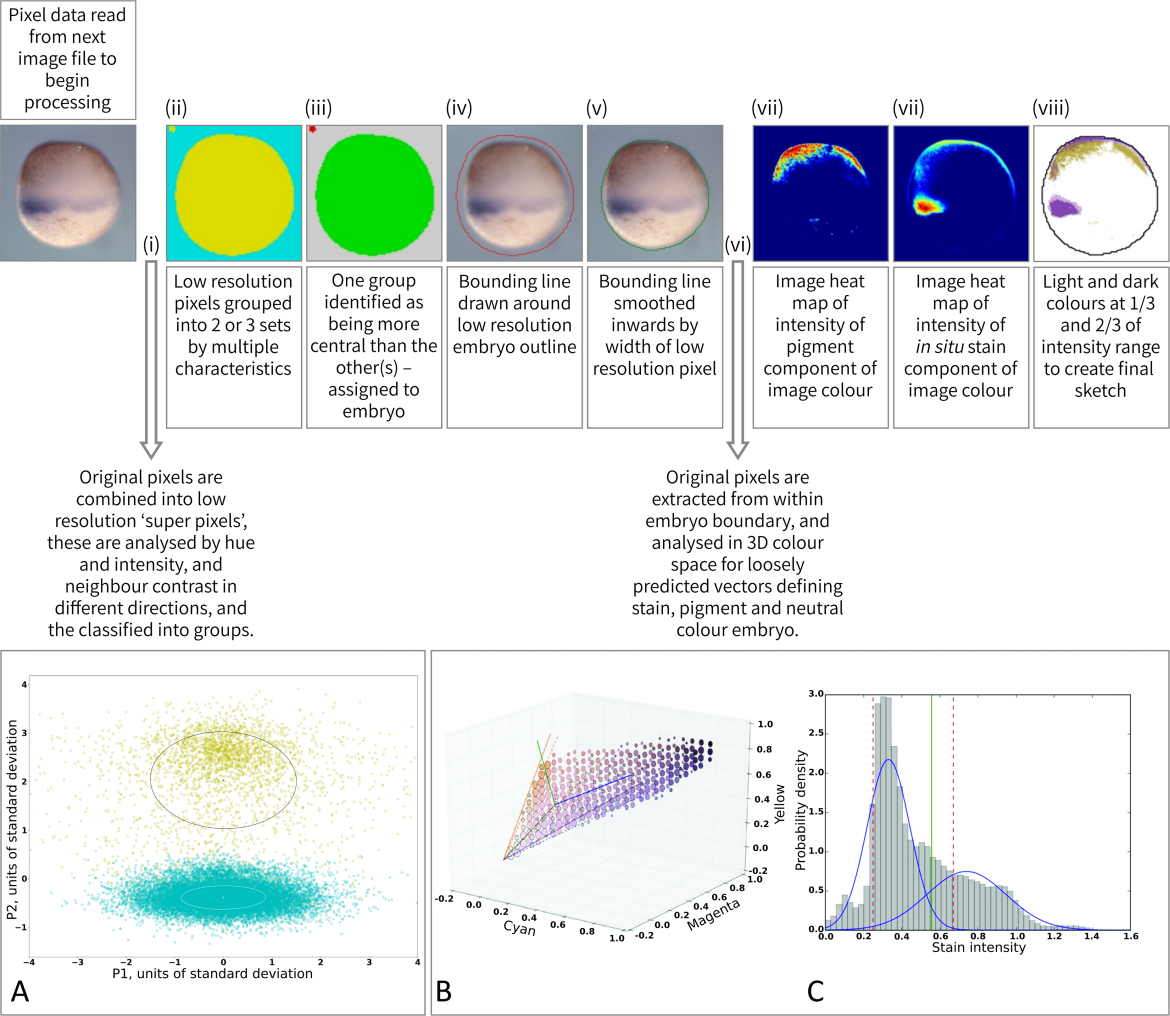
Overview of image analysis pipeline. (Upper panel) schematic representations of the stages of image analysis. Text boxes contain brief descriptions, see text for more detail, roman numerals correspond to steps in the workflow. Arrows show where data is extracted from the image for analysis. (A) Orthogonal projection of whitened 18 dimensional data extracted from the image. Colouring is made on result of clustering, with crosses and ellipses represent centres and covariances of the identified clusters. (B) Example representation of pixel colour density in the 3D colour space, showing identification of vectors corresponding to *in situ* stain, pigmented and un-pigmented embryo, used to identify regions of the embryo expressing the gene in question. (C) Example histogram of stain distribution. Data modelled as mixture of two Gaussians. The threshold is the smallest of mu + 2*sigma of the two components; it is represented as a solid green line. Dashed red lines represent range of values [.25, .67] the threshold is allowed to take.

**Fig 2:**
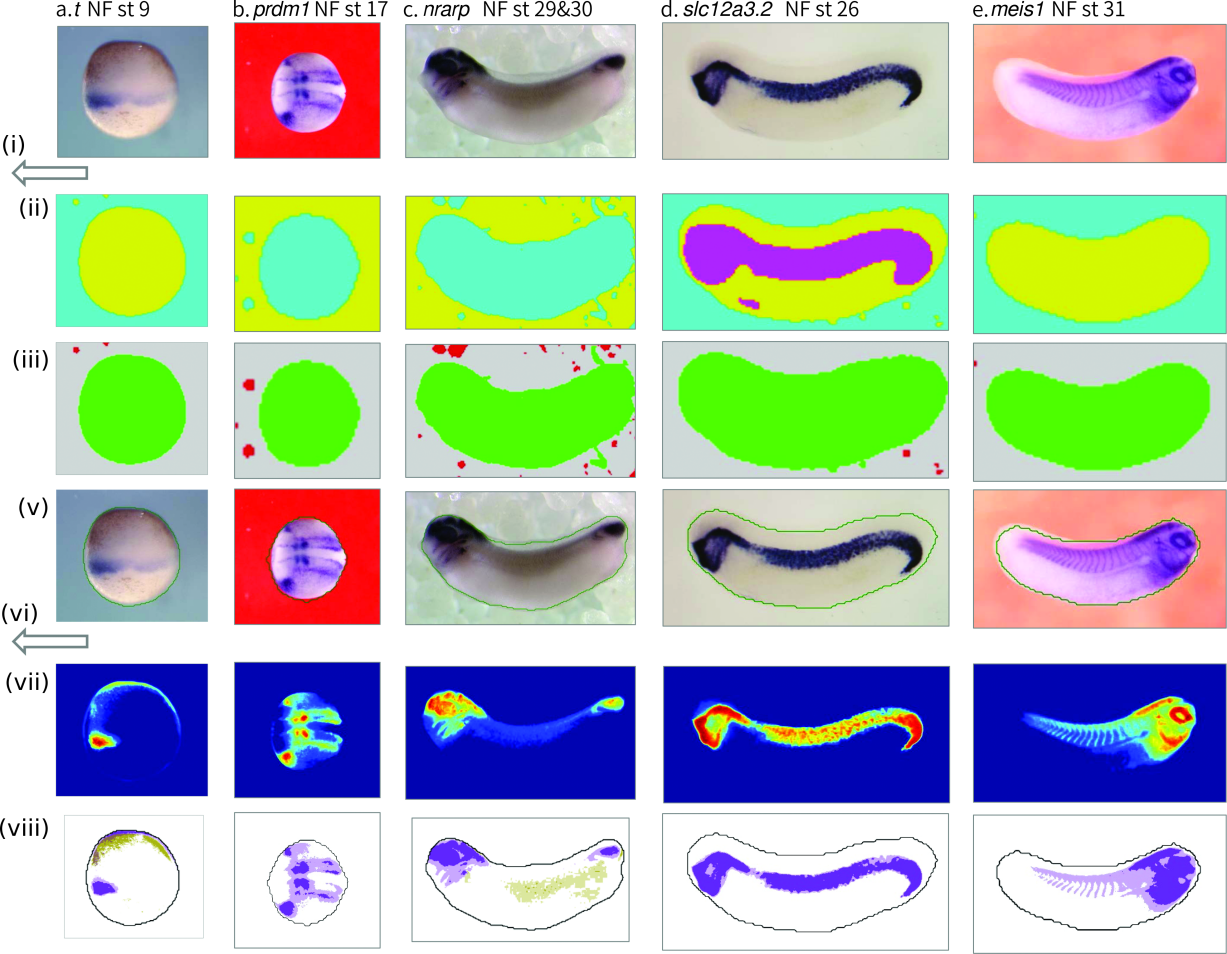
Graphical depiction of image analysis workflow for selected images with different shape embryos and a range of different background colours and textures. Images show that embryo detection and stain colour analysis is effective independently of a wide range of variation in image background and embryo characteristics. All images were analysed without changing initial parameters. Note image (d) where even the human eye struggles to distinguish the upper border of the embryo from the background.

Images are first processed to remove potential illumination artefacts and then downscaled. The degree of downscaling depends on the image, but is usually between 4- and 32-fold. Colour content and context are analysed for each downscaled pixel, and modelled as a mixture of either two (un-cleared images) or three (cleared images) Gaussian distributions. Pixels are assigned to the most likely distribution, and the image is mapped accordingly. The spatial distribution of each set of assigned pixels over the image is then considered: if a component is spread more uniformly across the image than other more compactly and centrally distributed component(s), then that component is considered to represent background. Isolated foreground regions that are small or close in colour to the background are re-assigned to the surrounding value. Embryo outlines are thus defined as the border between the background and other regions. Then the embryo outline is smoothed and moved inwards by the width of the low resolution pixels used at this stage. A detailed technical description of these processes can be found in Methods §2, and also see Figs 1 and 2 for illustration and examples. The embryo outline and its bounding box, with sides parallel to the image edges, are recorded with the image data, and a flag is set if the embryo outline touches the edges of the image.

### In situ staining pattern extraction

This algorithm determines the most likely hues within the previously detected outline of the embryo for stain and pigmentation. Analysis of the colour distribution inside the embryo outline is used to find statistically independent colour components of each image. These components are compared to the (detected) background colour (as bleed-through is possible), and likely stain and pigment colours: stain is assumed to be relatively blue-green and pigment relatively red-brown (see Methods §3). Heat maps of the determined stain and pigment colours are extracted from the data using adaptive thresholding, and overlaid on the image (Fig 1 and Fig 2). A score representing the degree of stain is associated with each image, and can be used to rank image for selection amongst sets of known duplicates (see Methods §6). This was useful in our analysis of the large, highly redundant, image collection (see below).

### Workflow outline

The overall image analysis workflow consists of 8 steps, these are summarised as follows (see also the visualisations in Figs 1 and 2):

- Pixel data in LAB colour space is extracted from image and transformed into lower resolution pixels.
- Low resolution pixels are classified on their colour and local roughness of the image and grouped into two, or three for cleared images, broad distributions corresponding to discrete areas of the image.
- Demarcated areas of image are assessed to determine the most likely to correspond to a roughly centrally positioned embryo or part embryo. The boundary is set at low resolution around the embryo.
- If the boundary touches the image border, the image is classified as a partial embryo.
- The boundary is smoothed to remove artefacts, and moved inward the width of one low resolution pixel to exclude background pixels close to the embryo.
- Pixel data is extracted from within the bounded embryo region of the image at native resolution, and analysed for distribution of hue and intensity in CMY colour space.
- Generalised prior knowledge (stain more blue/green than pigment, pigment towards red and darker than un-pigmented embryo) is used to identify likely vectors in colour space for *in situ* stain and pigment).
- The embryo is marked up according to areas of strong and moderate stain and pigment. Annotated version of image produced in register with original image.

### Validation

To validate the performance of our algorithms we used visual inspection of significant numbers of images selected by random sampling from our available collections. We would have preferred a purely computational method, but to our knowledge there are no suitable data sets of manually marked up in situ images of *Xenopus* embryos available. The closest available images were the manually marked up images from the XenMARK project[9], where the stained regions had been ‘registered’ by eye with, and transferred to, the embryo model diagrams. Even had we solved the registration problem for these embryos to enable a computational comparison with these data, we note that the subjective judgement applied during the XenMARK annotation process, as to the presence and limits of stained regions, would be much the same as using an expert annotator to compare side-by-side images of stained embryo and extracted stained regions. We further note that at this stage we were validating the correct identification of *in situ* stained regions within the images, irrespective of our understanding of their anatomical location.

We therefore validated our algorithms in two ways: quick visual inspection of two thousand images after steps (iii) & (iv) (see Outline Workflow above) to check correct identification of the embryo, as opposed to the background, within the image; and more intensive inspection of two hundred images after final mark up at step (viii) by WISH experts for correct interpretation of the *in situ* stain within the embryo.

The quick tests assessed three characteristics of the segmentation process: whether the image background component was correctly identified, whether the selected connected region corresponded generally to the embryo, and whether intersections of the frame edge with the embryo were correctly identified. For these tests we randomly sampled 1000 images from our local hosting of the XenMARK database, as well as 1000 images from the Ciau-Uitz/Patient collection. The errors observed were sufficently distinctive as to be effectively non-subjective, and tests results were scored true or false, with false positive and false negative results distinguished for the embryo/image boundary collision test. Most of the tests were passed at well over 99%, with the exception being for embryo/image edge collisions where false positive results were around 5%, depending on which collection images were from. These data are presented in more detail, along with expected error rates and corresponding 95% confidence intervals, in Table 1.

**Table 1:** Validation of image classification and embryo detection algorithms: Identification of non-subjective errors through visual inspection of 2000 randomly selected images. Image classification: was the image correctly classified as cleared or un-cleared? Background segmentation: was the background component identified correctly? Embryo region selection: did the connected region selected correspond to the embryo? Incomplete embryo detection: were embryos touching the image edge correctly identified as such?

Test	N	Errors	Expected error rate	Error rate CI_95_
Images from XenMARK				
Background segmentation	1000	3	0.004	[0.001, 0.009]
Embryo region selection	997	2	0.003	[0.0006, 0.007]
Incomplete embryo detection ^a^	995	59 / 3	0.06 / 0.004	[0.05, 0.08] / [0.001, 0.009]
Images from Ciau-Uitz/Patient collection				
Image classification	1000	0	< 0.001	[3.x10^-5^, 0.004]
Background segmentation	1000	1	0.002	[0.0002, 0.006]
Embryo region selection	999	1	0.002	[0.0002, 0.006]
Incomplete embryo detection ^a^	998	24 / 0	0.025 / 0.001	[0.02, 0.04] / [2.x10^-5^, 0.004]

The more intense inspection by two WISH experts looked at the precision of identification of the embryo outline and the extent of both the stained and pigmented regions within the embryo. The 200 tested images were randomly sampled from both the local XenMARK images and the Ciau-Uitz/Patient collection in proportion to the numbers of images in each collection. Each expert assessed the same set of images, but were instructed not to compare notes during this process. The experts were presented side-by-side with the original and marked up images and asked to give a subjective assessment of *good*, *intermediate* or *bad* for each of the three criteria: embryo outline, stained region and pigmented region. For subsequent analysis these assessments were converted to scores of 1.0, 0.5 and 0.0 respectively. These data are presented in Table 2.

**Table 2:** Validation of the algorithm output. Two WISH experts marked each of 200 processed images as *good*, *intermediate* or *bad* (1.0, 0.5 or 0.0) on three potentially subjective qualities: whether the embryo outline had been captured correctly, whether the stained regions were delineated correctly, and whether the pigmented regions were delineated correctly. For each quality we correlated the experts’ scores over all 200 images.

	Correlation	average score		good		good + intermediate	
		Expert 1	Expert 2	Expert 1	Expert 2	Expert 1	Expert 2
Outline	0.71	0. 8375	0. 805	79.5%	71%	88%	90%
Expression pattern	0.53	0. 8275	0. 6325	70.5%	36.5%	95%	90%
Pigment	0.62	0. 765	0. 535	68.5%	50.0%	84.5%	57%

Overall the results were encouraging, with both experts rating the algorithm for outline detection and expression domain extent (stained region) as close to or better than 90% in the good or intermediate categories. However it is quite notable that the correlation between the experts’ individual converted numerical scores was only a little over 0.5 for stained regions and 0.6 for pigmented regions. The underlying cause of this apparent discrepancy is likely in the different interpretation of the terms *good* and *intermediate* between the two experts, with Expert 1 being consistently more generous at the intermediate/good boundary then Expert 2. These results underscore the general problem in converting the variable intensity of the stained region into a computationally tractable expression pattern. We address this problem in part by providing a two-tone scale for mark up *in situ* stained regions. The pigmented region generally scored worse than the other criteria, although this is obviously of lower concern. We suspect, but have not shown in detail, that the dissimilarities in scores by the experts were attributed to their different assessment of the impact of artefacts caused by imaging conditions on the extracted pigment patterns. One of the more obvious artefacts affecting annotated stain pattern occurred in images where the embryo was illuminated from one side. In these cases the algorithm tended to interpret darker areas caused by shadow as more intensely stained.

### Application of the image analysis algorithms to a large image collection

To test the effectiveness of the algorithms, and to give us the opportunity to produce coherent sets of time dependent gene expression images, we applied them to a highly redundant image collection comprised 33,289 individual *in situ* images of *Xenopus laevis* embryos. These represented expression of 548 genes over the classical Nieuwkoop & Faber developmental stages [13], mostly between NF stage 6 (32-cell stage) and NF stage 50 (late tadpole stage), with an approximate 10-fold redundancy at each genes and development stage. We refer to this image set in the text as the Ciau-Uitz/Patient collection after its originators (AC-U and RP). This collection has been described previously [14], as have the methods by which they were produced[15]. The images from this collection used to illustrate our method have not been previously published.

**Fig 3:**
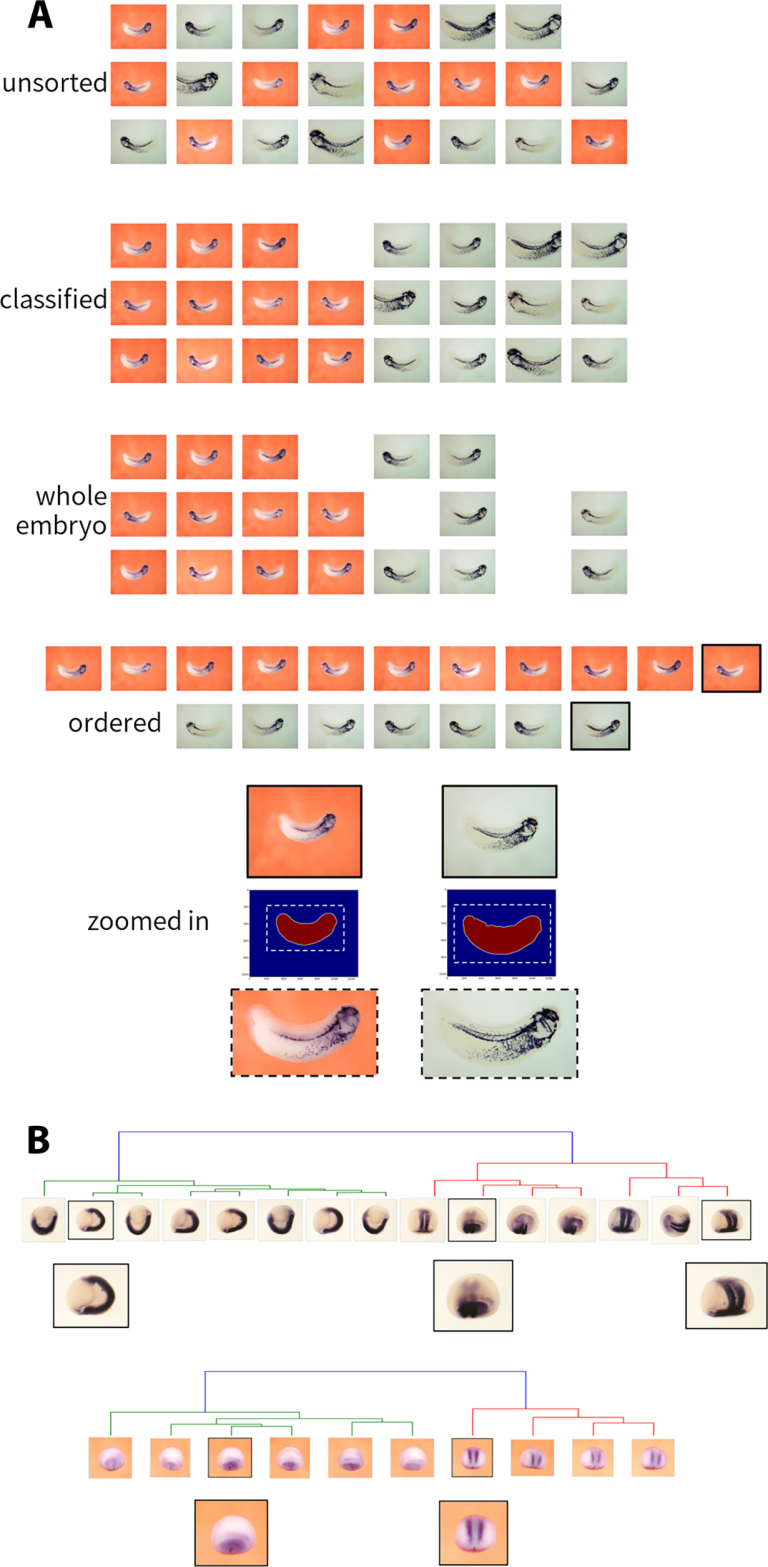
Rational selection of representative images to reduce redundancy. Unsorted images from a large collection for a given gene/development stage are first classified into cleared (grey background) and un-cleared (orange/red background) images. Embryo boundaries were detected within the image and embryo pixel colours analysed to yield predicted *in situ* stain, pigmentation or unmarked embryo. Embryos with predicted outline touching the image border were excluded (unless the outline in all images in the group touched the border). Images were sorted within groups by stain content for selection, and cropped for display where needed. (A) Lateral view images, NF stage 20+. (B) Quasi-spherical development stages (up to NF stage 20): images are clustered according to expression pattern similarity under rotational and other transformations (see also Fig 4), and the most stained image is selected from each well-separated group.

The collection had been pre-screened by one of the originators (AC-U) to retain only images of stages with clearly detectable gene expression, and in total the collection contains 2781 gene/stage groups. Embryos had been imaged either directly after histological staining, or after additional treatment with a clearing agent. In general, both types of preparation were available for each gene and stage. Un-cleared embryos had been imaged against an orange/red background, and cleared embryos against a grey background. All images were whole-mount, and although the majority of the images included the whole embryo, almost a third of the images contained close-ups of specific regions of the embryo. Images generally had associated meta-data, notably the gene name or probe/sequence ID and the developmental stage, all embedded in the name of the image file. Nearly all the Stage 22 and later images were lateral views; early stages included mixture of views. See Fig 3 for a visual depiction of the problem and its resolution.

Our aim was to reduce redundancy in this collection by extracting single representative whole-embryo images for each gene in the collection at each developmental stage, and for the cleared and uncleared embryos. In addition, for the earlier quasi-spherical embryonic stages, we also wished to select images to represent the different anatomical views of the embryo and expression patterns.

To achieve these ends we needed two additional algorithms: the first of these simply classifies the images into cleared and un-cleared on the basis of their statistical distributions of pixel colours, whilst the second uses image similarity clustering to identify different views of the spherical stage embryos using the previously detected *in situ* stain patterns. These algorithms are described in outline here, and more detail is given in Methods §4 and §5.

### Images classification to separate cleared and uncleared images

This algorithm classified the Ciau-Uitz/Patient images into two groups, those with un-cleared and those with cleared embryos. These had been consistently photographed against an orange/red background or a grey background respectively. This knowledge was used to sort the images on the basis of the statistical properties of the distribution of pixel colour in LAB space within each image, using a Gaussian mixture approach. See Methods §4 for details.

We found 18,254 un-cleared images, 15,034 cleared images, and 1 image was rejected as un-classified. Classification was important (a) to allow selection of both cleared and un-cleared images for each gene/stage where both were present, and (b) to allow a mixture of either two (un-cleared) or three (cleared) Gaussian distributions for the embryo/background analysis.

### Images clustering on expression pattern

**Fig 4:**
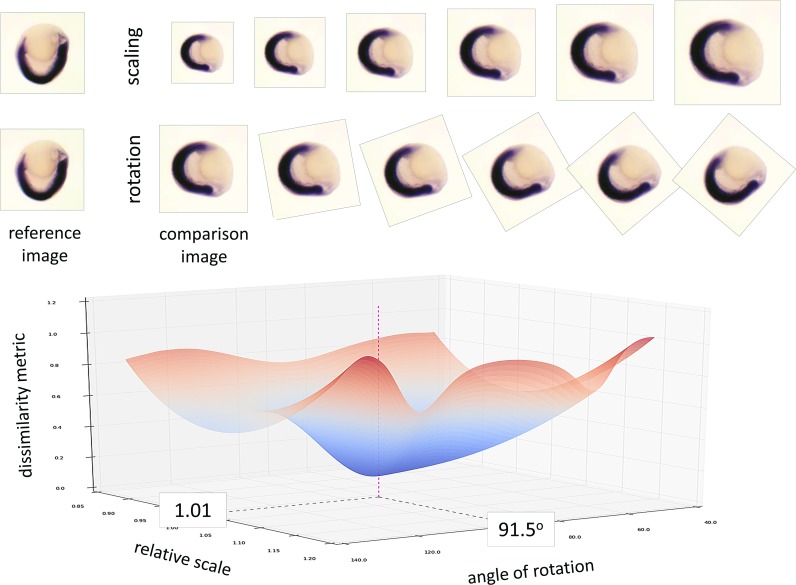
Simplified example of spherical stage image similarity clustering. Early development stages are routinely photographed from different directions to maximise information about the expression pattern. Reference and comparison images for the same gene and stage are compared under multiple transformations (scale, rotation, shear) to identify the set of transformations that minimises their dissimilarity. Here we see that a 91.5^o^ rotation and a 1.01 scaling suggest the most likely transformation between these two images. All images from the same gene and stage are compared with each other to identify images from (probably) the same view-point. See also Fig 3B.

The algorithm assesses similarity between expression patterns, and clusters images into groups, ideally representing different orientations of spherical embryos when photographed from different angles. Image comparison is performed after discovery of the embryo boundary and mark up of stain regions: within a group of same (spherical) stage images, each image is compared to all others by finding the combination of relative shift, rotation, scaling and shearing that maximises the overlap of stained regions. The minimum discrepancy achieved between two images is used as a *dissimilarity* metric, and pair-wise dissimilarities are used to perform clustering, of which the sub-groups represent the diversity of views of the expression pattern (Methods §5 and Fig 4). This algorithm represents a possible first step towards resolution of the image registration problem, although we take it no further in the current work.

### Validation of classification and clustering

We tested the image classification method using the quick visual inspection described above. We used the same set of 1000 images randomly selected from the Ciau-Uitz/Patient collection, and compared the predicted classification (cleared/un-cleared) with our observations. The image classification tool made no errors. These data are presented in Table 1.

In addition to this, we also assessed the expression pattern clustering performed with spherical stage embryos from the Ciau-Uitz/Patient collection. We had earlier noted that 79 image groups had embryo orientation information embedded in the image file names; this had not been used to support the computational clustering. We therefore compared the partitioning of the images by clustering to the partitioning provided by the image name annotation (see Methods §8), finding the sensitivity and the specificity of expression pattern clustering to annotated orientation to be 60.5% and 74% respectively. This lower sensitivity is primarily caused by non-informative expression patterns (i.e. uniform staining or absence of it) in some embryos, compounded by some imprecision between the described and likely actual viewing angles. On the other hand, the specificity value is explained by imperfect clustering of intrinsically variable expression domains and stain intensities.

### Image selection pipeline

The distinctive step for this analysis is to take any group of similar images and rank them according to the extent of in situ stain detected, from which a representative image can be easily selected (Methods §6 and Fig 3A).

The image selection pipeline runs as follows: (a) images are first classified as un-cleared or cleared to determine whether the initial image analysis needs to use two or three Gaussian components; (b) images are then analysed using the primary algorithms (described above) for embryo outline and *in situ* stain, recording whether the embryo touched the image frame or not, the position of the embryo outline and its bounding box, and the location and overall amount of *in situ* stain; (c) images are sorted into groups according to gene and developmental stage information; (d) images of spherical stages embryos are further grouped by anatomical viewpoint by clustering on the *in situ* stain patterns; (e) images within each final group are then ranked by in situ stain content to enable selection of one representative image, in our case the one with most stain, and selecting whole embryo images ahead of partial ones; and finally (f) images where the embryo was rather small are cropped to +15% of the embryo outline bounding box for display purposes. These functions are all provided within the command line Python program ‘select_images’, included in the ‘isimage’ module described below.

Application of the ‘select_images’ program (based on an earlier but fully functional version of the ‘isimage’ module) to the Ciau-Uitz/Patient collection resulted in the selection of 4,852 images, suitable for immediate web display, from the original 33,289 images. This smaller set was submitted to Xenbase, and is displayed on the appropriate gene pages. This effective consolidation of the original collection would have been extremely difficult to achieve by any other method.

**Fig 5:**
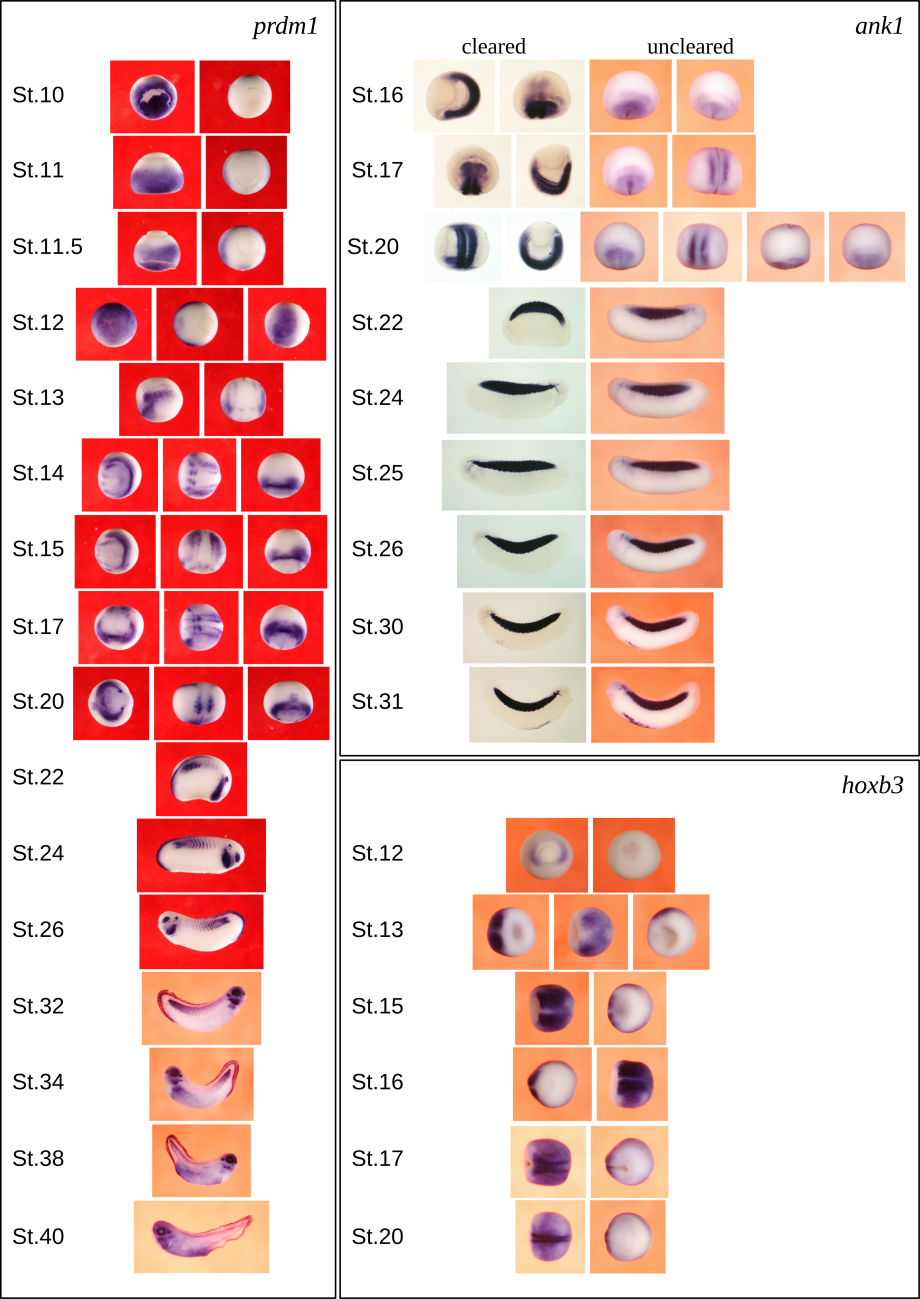
Developmental progression of expression for selected genes *prdm1*, *ank1*, and *hoxb3* during *Xenopus* embryo development. This is the result of applying our suite of image analysis tools to (in this case) the 334 original images for these three genes, and reducing them to a representative set of 65 images, including multiple views of expression patterns from the early spherical stages (pre-Stage 22).

To illustrate the power of this analysis to organise this large pool of data, showing the evolution of gene expression patterns during development, we include the selected images for the developmentally important genes *prdm1*, *ank1* and *hoxb3* (Fig 5). This illustrates well the importance of separating the spherical stage images by orientation, giving a clear picture of the intricate gene expression patterns developing through gastrulation and the setting up of neural patterning. To view the non-redundant version of the Ciau-Uitz/Patient image collection go to the Expression Search page in Xenbase, enter Patient Lab in the Experimenter field and click on the Search button.

### Algorithm summary and code availability

The two main algorithms, for embryo outline detection and stain/pigment decomposition, are the backbone of our image analysis suite and form the primary image analysis workflow described above. The image clustering algorithm was developed as a useful tool for grouping expression patters for early stage embryos, but is also a potential step towards image registration. These algorithms are implemented as parts of a Python module 'isimage’, including the program ‘analyse_image’ which provides access to the algorithms from the command line and allows expression pattern extraction to be performed on a per image basis. Code for these algorithms is made freely available on https://pypi.python.org/pypi/isimage/.

## Discussion

We have presented a general framework for analysis of whole mount *in situ* hybridisation images in *Xenopus* which is based on two specific advances. The first advance is to base segmentation around a novel method for building a statistical model of the image based on analysing colour and colour gradient in separate scales. The second advance is in the separation of *in situ* stain and pigment colouration using a hint based method taking in the prior (per image) determination of likely background colour in the segmentation step.

For the first advance, we have introduced an approach for unsupervised building of the explicit statistical model of the image background. It is based on capturing both colour and colour gradient in two scales and then using Gaussian mixture analysis to find the best separation of segments having different properties. The spatial distribution of segments is then analysed, and the background model is selected. Finally, the resulting boundary is smoothed employing re-normalised probabilities under the background model as the external force in the curvature minimizing PDE.

The novelty of this segmentation algorithm is in the way it utilises colour, spatial and edge information. This is in contrast to existing general purpose image analysis algorithms [16,17], which augment colours with spatial information, using edges as external constraint. Here we have jointly modelled the colour and the colour gradient, thus incorporating edge information into the GMM, whilst spatial distribution of pixels is used downstream to the GMM to classify the Gaussian mixture components. This approach was motivated by the known difficulties encountered by edge detectors in whole mount *in situ* images: (i) finding the correct transition between background and unstained regions at the edges of cleared and half-transparent parts of un-cleared embryos, and thus missing the correct outline[18]; and (ii) when the embryo is imaged against a feature rich background (for instance, and commonly, crushed ice), and the given approach detects spurious segments in the background[19,20].

Joint modelling of the colour and texture cues brings our algorithm closer to the texture classification method published by Permuter and colleagues[21,22]. This is, however, not completely suitable for *in situ* images because wavelets (employed in their approach) capture the texture at all scales [23], whereas in segmentation of *in situ* images variation smaller than a certain scale is unlikely to be significant. Thus the colour and the gradient data modelled by the GMM in our approach were captured in two consecutive layers of the Gaussian pyramid, ensuring that only significantly large texture elements are captured. Such an approach allowed us to put an upper limit on the number of Gaussian mixture components: in un-cleared images the model consisted of 2 components representing the embryo(s) and the background, and in cleared images no more than 3 components were considered, representing staining, the unstained embryo body and the background.

For the second advance, we have suggested a hint based method for pigment/stain separation and subtraction of the background colour, corresponding to bleed-through, from the embryo region of the image prior to analysis. The algorithm estimates the number of independent colours in a masked embryo image based on information theoretic considerations. Then, by employing FastICA algorithm and colour hints provided (primarily that stain is relatively blue-green and pigment relatively red-brown), it estimates and classifies stain and pigment colours. These hints would be configurable for application to other systems.

Our image analysis framework allows the determination of an object’s outline (i.e. the embryo’s outline) in an image, with minimal assumptions about background and object properties. It also allows the extraction of stain patterns from the image whilst excluding natural pigmentation from consideration. The quality of the analysis was independently checked by two WISH experts, who found it performed well. In addition, application of these tools allowed us to reduce redundancy in a set of 33,289 *Xenopus* embryo WISH images, resulting in 4,852 high quality representative images, making this collection amenable to display in a public resource. Analysis of the image collection, including pattern clustering where needed, took around 12 hours on 40 compute cores. The framework is published as a Python module ‘isimage’, and includes the image selection pipeline and a command line utility to extract the expression pattern from the image.

The significance of our approach to the initial segmentation of the image is well illustrated by its ability to find the embryo boundary in a wide range of image background colours and textures, not to mention variation in the shape, position and orientation of the embryo (seen clearly in Fig 2). This makes it potentially an ideal tool for retro-analysis of existing image collections, and stands in contrast to some of the earlier successes in the field which relied on controlling aspects of the image appearance such as background colour or texture compared to the embryo[3,5], effectively tuning the performance of their algorithms towards the images sets for which they were developed. We believe that our approach has great promise for the development of a more widely applicable tool set.

Our choice of manual validation at different steps in the image analysis pipeline was driven by a number of considerations, not the least of which was the availability of WISH experts to assess performance. In addition, we had some concerns about the potential for unconscious bias in the construction of a gold-standard reference set of manually annotated images, especially in the delineation of *in situ* stain regions. It is clear that notional boundaries of stained regions are often poorly defined as strong staining shades gradually into weaker and unstained regions, and that subjective judgments of these are inevitably made even by experienced annotators. This might be self-fulfilling if these image sets were constructed by ourselves, or lock the algorithm onto a particular operator bias, producing results with which other experts might not agree. The potential for different interpretation we saw clearly within our own experts and their judgment of how well the stain and pigment recognition algorithm worked. In the absence of a suitable gold standard we felt it was more effective to understand the actual performance of our algorithms, improving them iteratively by studying their behavior, and ultimately allowing other experts to assess their effectiveness. Nevertheless, we suspect there may be room for improvement, and are keen to put these codes into the public domain where others may build on our ideas.

A computational approach to validation was used in a recent paper describing image analysis in *Drosophila* [24]. They randomly sampled 200 *in-situ* images and tested the performance of their segmentation and registration algorithms against manually segmented and registered embryos. This may have been important, given the inclusion of the more complex registration step, and given that both segmentation and registration are (presumably) less prone to subjective variation in manual operations than *in situ* staining.

The primary weakness of the method is in the colour identification of pigmented regions of the embryo, and a tendency to be affected by brightly illuminated or shadowed sections of the embryo, which may say as much about the limitations of digital imaging under extremes of contrast. In this sense, our project has some clear pointers for optimising image generation where it is likely to be associated with subsequent computational analysis: notably avoiding bright and non-uniform illumination, and shadows. The difficulty in correctly identifying the extent of pigmented regions is less of a problem, as we are primarily interested in mapping the *in situ* stain; but we do believe that mapping the pigmented areas independently ensures that stain identification is more robust. In future work we will turn to the problem of registration, where we hope to be able to use models of known regional expression in combination with a refinement of our image comparison methods based on transformations of position, scale, rotation and shear to identify the likely anatomical viewpoint.

## Methods

### 1. Toolset for *in situ* image analysis

The algorithms developed were implemented as a set of functions and classes in the programming language Python. The code is based on numpy, scipy, sklearn, OpenCV libraries and organised in Python module ‘isimage’ in a way that allows use of either the individual algorithms or the image selection pipeline as a whole. The code can be freely downloaded from https://pypi.python.org/pypi/isimage/.

### 2. Unsupervised image segmentation

For each image in the LAB colour space, a Gaussian pyramid [25] is constructed ${\left\{I_{k}(x,y)\right\}}_{k=1}^{n}$; where *n* is the number of layers in the pyramid and *I*_*k*_:*R*^2^→*R*^3^ is the *k*-th layer of the pyramid. Search for an object is performed simultaneously in two adjacent layers of the Gaussian pyramid. The largest dimension of the biggest layer used is less than 200 pixels. From each of the two layers, colour and edge information are extracted. The edge information is represented as partial derivatives $\frac{\partial }{\partial x}I_{k}(x,y)$ and $\frac{\partial }{\partial y}I_{k}(x,y)$ computed with the Scharr operator [26]. The data extracted from the low-resolution layer are interpolated to match the high-resolution layer dimensions, with the same Gaussian kernel used for the pyramid creation. The information from both layers is combined resulting in 18 parameters for each pixel.

The data is then ‘whitened’ and only informative principal components are used in the subsequent analysis. Principal components whose singular values, divided by the sum of all singular values, exceeded 10^−6^ are considered informative.

To learn the borderline between the background and the foreground, the data is modelled as a mixture of Gaussian distributions. Since in un-cleared images the embryo is very distinct from the background, those images are modelled as the mixture of up to two Gaussians representing an embryo and the background. On the other hand, in cleared images the difference in colour and texture between unstained parts of embryo and the background can be subtle, compared to their difference from stained regions. In these cases, the two-component mixture will often draw the line between stained and unstained areas of the embryo thus counting unstained regions as the background. To handle this, cleared images are modelled as having up to 3 components, with an assumption that background is captured in one component and the embryo is captured in two other components. The actual number of components is determined with Bayesian information criterion[27]. The GM model was fitted using a random sampling of the image, but excluding data within three pixels of the image edges. GM fitting and component classification is repeated three times, then the most likely foreground/background decomposition is brought forward for further analysis.

Once the model is fitted and pixels were assigned to one of the components, there is a need to classify the components themselves as representing either the object or the background.

$$X_{i}=\left\{(x-\frac{w}{2},y-\frac{h}{2});\;(x,y)\in C_{i}\right\}$$

Here *X*_*i*_ is the set of pixels of the image *I*, which is classified as belonging to a component *C*_*i*_. *w* and *h* are the width and the height of the image respectively.

To choose the most likely classification of the components, a Bayesian model selection approach is used:

P(M|X)∝P(X|M)P(M)

$$P(X|M_{j})=P(X_{i=j}|M_{j})P(X_{i\neq j}|M_{j})$$

Here *M* is a random variable representing a model and *M*_*j*_ is the model in which the j^th^ component is believed to be the background. The prior distribution of the models was uniform.

Based on the assumption that the embryo resides in the middle of the picture, background pixels are modelled to be distributed uniformly across the image.

$$X_{i=j}|M_{j}∼Uniform(\left[0,w]\times [0,h\right])$$

$$P(X_{i=j}|M_{j})=\frac{1}{wh}$$

And object pixels are modelled to be distributed normally around the centre of the image.

$$X_{i\neq j}|M_{j}∼N(0,\Sigma )$$

$$P(X_{i\neq j}|M_{j},\Psi ,\nu )=\int N(X_{i\neq j}|0,\Sigma )W^{-1}(\Sigma |\Psi ,\nu )d\Sigma =\frac{{\left|\Psi \right|}^{\frac{\nu }{2}}\Gamma_{2}(\frac{\nu +n_{j}}{2})}{\pi^{n_{j}}{\left|\Psi +X_{i\neq j}X_{i\neq j}^{T}\right|}^{\frac{\nu +n_{j}}{2}}\Gamma_{2}(\frac{\nu }{2})}$$

Here *N*(**0**,***Σ***) is a two-dimensional normal distribution with zero mean and ***Σ*** covariance matrix. *W*^−1^(***Ψ***,*ν*) denotes the inverse Wishart distribution, the conjugate prior to multivariate normal with known mean. The parameters of the prior distributions ***Ψ*** and *ν* were chosen to be *ν* = 1 and $\Psi =\left[\begin{array}{cc} {\left(\frac{w}{2}\right)}^{2} & 0\\ 0 & {\left(\frac{l}{2}\right)}^{2} \end{array}\right]$. ***Γ***_2_ is the multivariate gamma function. *n*_*j*_ is the number of pixels which belong to the foreground under the j^th^ model.

If none of the models is substantially better, the component whose pixels are present the most often at the image edge is classified as the background.

The assumption is that the image contains only one embryo, but the foreground found above, along with the embryo outline, will have some noise in the form of a number of disconnected islands. In order to remove the noise in the foreground, a connectivity graph is created by connecting adjacent pixels belonging to the foreground. Disconnected sub-graphs of the connectivity graphs are extracted by a spectral graph theory approach[28]. The graph is recursively cut at points where the sorted elements of the eigenvector associated with zero valued eigenvalue of the Laplacian matrix of the graph exhibit the biggest jump exceeding the threshold 10^−4^.

The sub-graphs are compared based on their size and the difference of the average colour from the average colour of the background. The island having the maximum product of the square root of its size and the difference from the background is selected as the embryo outline.

$$Embryo\;outline=\underset{S_{i}\in S}{\max}\left(\sqrt{\left|S_{i}\right|}\|M_{S_{i}}-M_{bg}\|\right)$$

Where *S*_*i*_ is a disconnected sub-graph of the connectivity graph *S*; $M_{S_{i}}$ is the average colour of pixels in sub-graph *S*_*i*_; *M*_*bg*_ is the average colour of the background.

The steps of splitting the foreground into disconnected components and selecting the most outstanding island are repeated twice, with the first round taking the entire foreground into account and the second round ignoring the parts of the image that are 3 pixels away from the image edges. The outline is considered touching the image edge if the selected foreground component touches the image edge in both cases. The embryos in the image are assumed to have no holes, thus all holes inside the closed contour around the selected foreground element are filled. In case the photographed embryo extends beyond the image, it is sometimes necessary to close the embryo contour along the image edge before filling the holes. To close the embryo contour along the image edge a randomly selected quarter of pixels in the 2-pixel band around the image edge is marked as foreground and then islands containing a single pixel are reverted to the background. The process is repeated until the size of the biggest foreground element increases by less than 5% during the last iteration, but no more than 100 times.

The resulting outline is smoothed by minimizing local curvature of the outline contour using the geodesic active contour framework proposed in [29]. The framework assigns to a curve an energy functional, which depends on the contents of an image. To minimize the functional [29] proposes a contour evolution partial differential equation (PDE), the stationary state of which minimizes the functional. The PDE contains three members on the right hand side; first corresponding to curvature force, which minimizes local curvature; second is balloon force, which tends to expand or contract the contour; third is the image attraction force, which makes the contour reflect the contents of an image. The stationary state of the equation is found using the level set approach as suggested in [29], with zero balloon force everywhere and 8 iterations for curvature force. Since the unsmoothed contour is the collection of points where the probability of belonging to the background under GMM equals the probability of belonging to the foreground, it is natural to use the probabilities as a base for the image attraction force; the log-likelihoods of pixels are first divided by the minimum log-likelihoods for the background and foreground respectively and then summed.

$$g(x,y)=\frac{logP\;((x,y)\in C_{bg}|GMM)}{\underset{x,y}{\min}\;logP\;((x,y)\in C_{bg}|GMM)}+\frac{logP\;((x,y)\notin C_{bg}|GMM)}{\underset{x,y}{\min}\;logP\;((x,y)\notin C_{bg}|GMM)}$$

Images with the outline touching the image edge are considered to be presenting incomplete embryo, and a flag is set in the image data to record this. Next, the outline is scaled up to match the original image dimensions. The resulting contour is then contracted by the number of pixels corresponding to half the scaling factor between the original image and the smallest layer in the Gaussian pyramid that is used for the embryo search, to make the final embryo outline. A bounding box is recorded in the image data, which is the rectangle with sides parallel to the images edges that just contains the embryo outline.

### 3. Stain distribution extraction

In the ‘select_images’ program there is a pre-processing step before stain distribution extraction, since differences in embryo illumination can affect the estimation of the stain intensity. Where appropriate, the background colour is assumed to be the same for all compared images (either cleared or un-cleared images) in a specific gene/stage group where the images are from the same collection. Thus any differences in background colour amongst those images are assumed to be caused by imaging conditions. The differences in average luminosity in all images to be compared are compensated. Then the images are processed independently.

An image is converted into CMY colour space. The model behind the analysis assumes that an image is “painted” with a small number of paints, with each pixel colour being a linear mixture of different amount of each paint:

$$x_{i}=As_{i}$$

Where *x*_*i*_ is a CMY colour of *i*^th^ pixel, *A* = [*a*_*s*_,⋯] is a matrix containing CMY values of each of the paints normalised to unit length as its columns, *s*_*i*_ is a vector representing amounts of each paints in the pixel.

One approach to find a solution to the equation is independent component analysis; here we use FastICA algorithm to find the independent components [30]. The algorithm solves the equation:

$$K(X-\bar{X})=MS$$

Where *M* is a symmetric *n*×*n* “mixing” matrix; *K* is a *n*×*m* whitening matrix; *m* is the dimensionality of the data; *n*≤*m* is a number of independent components; *S* is latent “source” variables.

Despite the speed and underlying assumptions of the FastICA algorithm aligned well with the needs of this project, the algorithm has some drawbacks. As seen from the above equation, the FastICA algorithm finds a solution up to a multiplicative constant; it rather finds independent axes in data since the signs or magnitudes of the independent components cannot be determined. Furthermore, since FastICA finds the solutions for mean-centred rather than for zero-centred data an independent axis would correspond to a spectrum of colours rather than to the single colour of the respective paint.

To get around these issues, prior knowledge of the expected colours of the paints is used. From the equation above, the maximum possible number of components cannot exceed the dimensionality of the data, three colour channels in our case. Thus no more than three colour components are expected: stain, pigment, and background where *C* = [*c*_*s*_,*c*_*p*_,*c*_*b*_]. The background colour is estimated by averaging the colour of pixels outside the embryo outline, whilst stain and pigment colours, blue and brown respectively, were the same for all images in Ciau-Uitz/Patient collection; all expected colours are normalised to unit length.

Not all of the three components will always be present in an embryo image, thus there is a need to estimate the actual number of independent components. The FastICA algorithm finds the solution by choosing such entries in *M* that minimize the normality of the distribution of “source” variables. From that, it appears natural to estimate the number of components by maximizing the average information content per component as measured by Kullback-Leibler divergence of the empirical distribution of a “source” component from the fit Gaussian distribution.

If the number of components does not match the number of expected colours, some of the expected colours are assumed not present in the image and are removed from the set. At this stage the stain colour is assumed to always be present, thus if the estimated number of components is one the expected stain colour is the only one left in the set. If the estimated number of independent components is two whilst the number of expected colours is three, there is a need to identify which colour is missing. Since it is preferable to detect faint stain, whilst faint pigment can safely be ignored, the assumption at this step is that stain colour is always present and the missing colour is either background colour or pigment colour.

The ambiguity is resolved by maximising the linear combination of the absolute values of the determinant of the correlation matrix between the distribution of the colours in the image and the distribution of independent “sources”, and the cosine between the normals to the planes formed by the vectors left in the set and two first principal components (principal plane) of the pixel colours.

$$C_{e}=\underset{C\in \{\left[c_{s},c_{b}],[c_{s},c_{p}\right]\}}{\mathrm{argmax}}\left\{\left|det[corr(T(X_{e}-\bar{X_{e}}),C^{+}X_{e})]|+10|\hat{\left(C_{0}\times C_{1}\right)}∙\hat{\left(T_{0}\times T_{1}\right)}\right|\right\}$$

Where *C* is a matrix containing in its columns n expected colours including stain colour. *X*_*e*_ is colour values of pixels inside the embryo outline. *T* = *M*^−1^*K* is matrix of independent components. $\hat{u\times v}$ means cross-product of *u* and *v* normalized to unit length.

If the resulting set of expected colours includes the pigment colour, the stain and pigment colours are adjusted. The adjustment is done by rotating vectors representing the colours in the CMY colour space around their mean by an angle ranging from -15 to +15 degrees in order to make the plane formed by the vectors as parallel as possible to the principal plane of the pixel colours.

Using both independent axes and expected colours, it is possible to estimate the components of *A*. The estimation is done in two steps: first, the proposed components of *A* are computed from the data and independent axes; second, the proposed components are compared to expected colours; the set of proposed components closest to the expected colours are accepted.

The computation of the proposed components is based on the assumption that the components of *A* should be as far as possible from the average colour. Thus, after picking an independent axis and choosing a direction from the mean, the proposed component then equals the normalised to unit length point on the independent axis where the projection of image pixels close to the axis in colour space is maximal. To increase robustness of the method to the colour imprecisions, only pixels sufficiently distant from white colour are used.

$$a_{i}^{\prime }={\left(\underset{x\in X;\|x\|\geq l_{\min}}{\mathrm{argmax}}\left\{\|{\left(x-\bar{X}\right)}_{∥z_{l}\hat{m_{k}^{*}}}\|∙e^{-\frac{{\left\|{\left(x-\bar{X}\right)}_{⊥z_{l}\hat{m_{k}^{*}}}\right\|}^{2}}{2∙{d_{max}}^{2}}}\right\}\right)}_{∥z_{l}\hat{m_{k}^{*}}}$$

Where *x* is the colour of a j^th^ pixel inside embryo outline, ‖*x*‖>*l*_*min*_, it is set to 0.15; $\hat{m_{k}^{*}}$ is the k^th^ column of M* = *K*^+^*M* normalised to unit length; *z*_*l*_∈{1,−1} is a direction with respect to $m_{k}^{*}$. Subscript notation *x*_∥*v*_ means the parallel and *x*_⊥*v*_ the orthogonal component of *x* with respect to *v* such that *x* = *x*_∥*v*_+*x*_⊥*v*_; *d*_*max*_ controls the effective distance of the pixel colours from the independent axis, and is set to 0.05.

The best set of proposed components is found by minimizing the weighted average distance between proposed components and the expected colours multiplied by the specificity of the match.

$$\hat{A}=\underset{A^{\prime }}{\mathrm{argmin}}\sum_{i=1}^{n}w_{i}\frac{{\left\|c_{i}-a_{i}^{\prime }\right\|}^{2}}{\sum_{j\neq i}\left\|c_{i}-a_{j}^{\prime }\right\|}$$

Where *A*′ is the proposed components matrix; *n* is the estimated number of independent components; $a_{i}^{\prime }$ is the i-th column of *A*′; *c*_*i*_ is the i-th column of *C*_*e*_; *w*_*i*_ is a weight associated with each expected colour, the weights reflected a prior confidence in that colour: *w*_*s*_ = 0.5,*w*_*p*_ = 0.05,*w*_*b*_ = 1. Since the background colour is computed from the image it has the highest confidence. There is less confidence in prior knowledge of the stain colour since it can vary due to imaging conditions, and choice of stain reagents. The argument behind the much smaller confidence level for pigment colour is two-fold; firstly, the colour can vary due to biological differences or imaging conditions. Secondly, the model used here assumes linear colour mixing whereas the imaging condition can produce saturation effects and hence non-linear colour mixing in lighter or darker parts of images, the number of independent components can be overestimated with the superfluous components being far from any of expected. The low weight for the pigment allows that false component to be associated with the pigment in case saturation occurs in the lighter part of the spectrum. In case the pigment component is associated with saturated stain, the estimation of *A* is done assuming no pigment component is present.

The estimated stain colour $\hat{a_{s}}$ is considered confidently estimated if the relative positive contribution of expected stain colour in $\hat{a_{s}}$ is over 5%.

$$\frac{max(0,\;q_{1})}{\sum_{i=1}^{n}max(0,\;q_{i})}>0.05;where\;q=C_{e}^{+}\hat{a_{s}}$$

The spatial stain distribution is found by solving the equation $X=\hat{A}S$ for all image pixels and taking the first “source” components.

‘Adaptive thresholding’ is applied to the stain distribution to make sure that only significant staining is taken into account. This is done by modelling the stain distribution inside the embryo outline as a mixture of two Gaussian distributions, one of which would represent ‘noise’ and the other would be considered the ‘signal’. The noise is filtered out by selecting a threshold so that 95% of noise is under the threshold. The threshold is range limited by the interval [0.25, 0.67] because values of staining/pigmentation below 0.25 level would be too close to white to be significant; on the other hand staining/pigmentation above 0.67 would be significant anyway, even if doesn’t form a pattern.

$$S∼\sum_{i=1}^{2}\pi_{i}N(\mu_{i},\sigma_{i})$$

$$t=\min\;\left(0.67,\;\max\;\left(0.25,\;\underset{i}{\min}\left(\mu_{i}+2\sigma_{i}\right)\right)\right)$$

Where *S* is the random variable representing staining; t is the threshold.

If the background colour has non-zero projection on the stain colour it results in the “background” noise. To remove the noise from the spatial stain pattern without creating sharp artefacts, a smooth mask is created from the embryo outline as follows. Mean and standard deviation of stain amount in the area outside the embryo outline are computed. The mean amount of stain inside morphological gradient of the embryo outline is computed and recorded. The embryo outline is eroded for one round. These two operations are repeated several times, or until the mean of the stain in the gradient band exceeds the mean plus two standard deviations of the stain in the background. Then a smooth mask is created with a sigmoid profile with the inflexion point located at the distance from the embryo outline where the mean amount of stain in the corresponding gradient band is at the minimum, or if a minimum is not reached, at half way to the maximum of the mean amount of stain.

### 4. Classifying images as cleared or un-cleared

In the Ciau-Uitz/Patient collection of 33,289 images, un-cleared images had orange/red backgrounds and cleared images had grey backgrounds. They were sorted into groups according to the colour distribution of pixels within each image. Initially, images were converted into LAB colour space, and pixel values accessed via standard library functions.

To capture the colour distribution of pixels in an image, means *M*_*i*_ and covariance matrices *C*_*i*_ were computed for each image. Components of the mean vector and lower triangular parts of the Cholesky decomposition of the covariance matrices were combined to produce a data point representing colour distribution in a particular image.

$$M_{i}=\langle m_{1},m_{2},m_{3}\rangle$$

$$L_{i}L_{i}^{T}=C_{i}$$

$$L_{i}=\left[\begin{array}{ccc} l_{11} & 0 & 0\\ l_{21} & l_{22} & 0\\ l_{31} & l_{32} & l_{33} \end{array}\right]$$

$$x_{i}=\langle m_{1},m_{2},m_{3,}l_{11},l_{21},\ldots ,l_{33}\rangle$$

To learn the best separation between cleared and un-cleared images, the distribution of the data was modelled as a 2–component Gaussian mixture [31]. The model was fit using the expectation maximization algorithm. As a result images were assigned to one of the two components, hence separating cleared and un-cleared images.

### 5. Clustering similar views

Embryo images, in a particular gene/stage groups with recorded stage earlier than 22, are clustered on their expression patterns, with cleared and un-cleared images clustered separately.

To find the distance between images, the spatial stain distribution of each image is aligned with those of all other images in the group. Images of stain distribution are normalized by the standard deviation of the pixel intensities and down-sampled so none of their sides exceeds 100 pixels. Alignment is done by minimizing a function with the squared Euclidian distance between spatial stain distributions of the images as the external energy with respect to linear-affine transformation of one of the images[32]. The distance is penalized for scaling. Minimization is done with BFGS algorithm.

$$I[A,b]=\iint {\left\|S_{T}(Ax+b)-S_{R}(x)\right\|}^{2}+\alpha {\left(ln({\left|A\right|}^{2})\right)}^{2}dx$$

Where *S*_*i*_(⋅) is the spatial stain distribution in an image; *α* is the regularization constant.

For each pair of images in the group, one of the images is taken as a reference and minimization is done from eight initial positions of the template image: 4 rotations by 90 degrees of original image and the same of a flipped image. Then the process is repeated with the other image taken as a reference. The minimum of the 16 minimal distance values is taken as the distance between the expression patterns.

All the pairwise distances taken with the minus sign formed a similarity matrix. The clustering is done by adaptive affinity propagation algorithm [33] with the number of clusters not exceeding four.

### 6. Ranking images on staining

In general, images are ranked on the 85^th^ percentile of the stain distribution and the image of the highest rank in the gene/stage group is selected. In case of clustered images of early embryos, the total similarity of an image in the cluster is added to 85^th^ percentile of the stain distribution to account for how well the image represents the cluster.

### 7. Computation of expected error rates and CI_95_

Expected error rates and corresponding 95% confidence intervals were calculated under the Bernoulli model with uninformative Beta(1, 1) prior.

### 8. Comparison of expression pattern clustering to embryo orientation annotation

Partition of the images by the clustering was compared to the partition by embryo orientation using Wallace pairwise agreement coefficient [34,35]. The Wallace coefficient from partition A to partition B is a ratio $W_{A\to B}=\frac{a}{\left(a+b\right)}$; where *a* and *b* are entries of a mismatch matrix $\left[\begin{array}{cc} a & b\\ c & d \end{array}\right]$, which in row 1 has the numbers of pairs in the same cluster and in row 2 the numbers in the different clusters of A, and in columns the same for the partition of B. In our case, the Wallace coefficient has the meaning of sensitivity of the clustering to embryo orientation, i.e. the proportion of pairs of images put in the same cluster that have the same embryo orientation. We augmented the clustering sensitivity by a clustering specificity coefficient $\frac{d}{\left(c+d\right)}$.

### 9. Parameter fitting

All algorithm parameters were tuned using manual procedure similar to cross validation, with a training image set that reflected image collection variability. The procedure consisted of recursively applying the following two steps until optimal parameter values were found. First, parameters were adjusted to allow the algorithm to perform best on a small subset of the training set containing images the algorithm performed worst at. Then the algorithm with parameter value found at the previous step was applied to the whole training set to assess the generality of the value.
